# Organ Repair, Hemostasis, and In Vivo Bonding of Medical Devices by Aqueous Solutions of Nanoparticles**

**DOI:** 10.1002/anie.201401043

**Published:** 2014-04-16

**Authors:** Anne Meddahi-Pellé, Aurélie Legrand, Alba Marcellan, Liliane Louedec, Didier Letourneur, Ludwik Leibler

**Affiliations:** Inserm U1148, LVTS; UniversitéParis 7, Université Paris 13, Sorbonne Paris Cité, Hôpital Bichat, 46 rue rue H Huchard, 75018 Paris (France); UniversitéParis 13, Sorbonne Paris Cité, Paris (France); Matière Molle et ChimieUMR 7167 CNRS - ESPCI ParisTech, ESPCI, 10, rue Vauquelin, 75005 Paris (France); Université Pierre et Marie Curie, Sorbonne UniversitésParis (France)

**Keywords:** bioadhesives, nanoparticles, scaffolds, surgery, wound repair

## Abstract

Sutures are traumatic to soft connective tissues, such as liver or lungs. Polymer tissue adhesives require complex in vivo control of polymerization or cross-linking reactions and currently suffer from being toxic, weak, or inefficient within the wet conditions of the body. Herein, we demonstrate using Stöber silica or iron oxide nanoparticles that nanobridging, that is, adhesion by aqueous nanoparticle solutions, can be used in vivo in rats to achieve rapid and strong closure and healing of deep wounds in skin and liver. Nanoparticles were also used to fix polymer membranes to tissues even in the presence of blood flow, such as occurring after liver resection, yielding permanent hemostasis within a minute. Furthermore, medical devices and tissue engineering constructs were fixed to organs such as a beating heart. The simplicity, rapidity, and robustness of nanobridging bode well for clinical applications, surgery, and regenerative medicine.

Stopping bleeding (hemostasis), preventing body fluid leakages, wound closing, and organ repair are everyday challenges in medical and surgical practice.[1] Sutures and staples are standard and efficient tools. Still, suturing can be demanding in inaccessible body regions or within minimally invasive surgery. Moreover, sutures are traumatic to tissues especially soft tissues such as liver,[2] spleen,[3] kidney,[4] or lung.[5] During last decades, synthetic or biological tissue adhesives that rely on in situ polymerization or cross-linking reactions have emerged as a complementary technique.[1c, 6] However, tissue adhesives currently available in clinical practice present significant inherent limitations such as toxicity, insufficient strength, and/or excessive swelling.[1c, 6c, 7] Biomimetic approaches and new chemistries that yield polymer materials with adaptable adhesion strength are under development.[6b,e, 8] In practice, gluing or sealing with polymers remains a complex process: it requires both stringent storage and preparation conditions before in vivo glue application or in vivo initiation and control of chemical polymerization or cross-linking reactions.

Recently, a novel approach to adhesion of hydrogels has been proposed.[9] It relies on the use of aqueous nanoparticle solutions in place of polymer adhesives. The method does not require a chemical reaction: a droplet of nanoparticle solution is spread on a gel surface and gel pieces are brought into contact. Nanoparticles that are adsorbed to gel surfaces act as connectors between the pieces and assure adhesion. The adhesion strength is brought by macromolecules of the gel that adsorb onto the nanoparticles. Under constraint, adsorbed layers are able to reorganize, dissipate energy, and prevent interfacial fracture propagation. The approach is not limited to synthetic hydrogels, and the adhesion was shown ex vivo for two slices of calf liver using a silica nanoparticle solution.

It is natural to extend the principle of adhesion by particle nanobridging to in vivo wound closure (Scheme 1). Nevertheless, decades of research on polymer tissue adhesives has shown how challenging it is to achieve an adequate adhesion in the presence of blood, and in particular within a short time compatible with clinical practice. Moreover, adhesive joints have to withstand after-closure constraints of in vivo conditions, such as tissue motion or body-fluid flow. Herein, we demonstrate the applicability of silica nanoparticle aqueous solutions to repair injuries in two types of tissues, namely skin and liver, in a rat model. We also show that strong and rapid wound closure and repair can be achieved with iron oxide nanoparticles. Iron oxide nanoparticles are metabolizable and, as an additional boon, they could provide a contrast in magnetic resonance imaging enabling clinical in situ observations.[10]

**Scheme 1:**
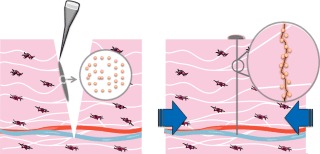
The concept of nanobridging for wound closure. Left: A droplet of nanoparticle solution is spread with a micropipette or a brush at the wound surface of a tissue. Right: The wound edges are brought into contact by gentle manual pressure (blue arrows). Nanoparticles adsorbed onto tissue components at the site of injury form numerous connectors that link wound edges together (inset).

Wound closure is not the only area of applications that could benefit from adhesion brought by nanoparticles. For example, hepatic resection has been increasing in frequency in the management of metastatic or primary neoplasms of the liver. Although mortality for this procedure has steadily decreased, the morbidity mainly associated with operative time and blood loss remains high, especially in cirrhotic patients. During hepatic resection, control of bleeding is a crucial problem faced by surgeons.[2, 5a, 7c, 11] We show herein that particle nanobridging can provide a means for rapid and permanent hemostasis after rat liver resection. To this end a polymer synthetic film was coated by nanoparticles by adsorbing nanoparticles onto its surface and spread to cover the intensely bleeding liver section. Strong adhesion and permanent hemostasis were achieved within a minute.

To illustrate possibilities of nanobridging to attach medical and tissue engineering devices to organs, we permanently fastened a 3D tissue-engineering scaffold to a beating rat heart.

To optimize adsorption onto tissue surface it is advantageous to avoid using nanoparticles that are stabilized by polymer layers. Indeed, grafted or adsorbed polymers can be effectively repelled by intercellular (macro)molecules and thus prevent adsorption of particles onto tissue surface. Thus nanoparticles that have been optimized to circulate in the body are to be avoided. Two types of nanoparticles were thus used in this study. Silica nanoparticles (SiO2NP) with radius of about 50 nm (Supporting Information, Figure S3) were synthesized by the Stöber method and applied as a solution in deionized water at concentration of 30 wt % (pH 8.5) or, when indicated, as a powder. Iron oxide Fe_2_O_3_ nanoparticles (Fe2O3NP) were purchased from Alfa Aeser, stabilized by citric acid, peptized, and used in aqueous solution in milli-Q water at 42 g L^−1^ (Supporting Information, Figure S4).

All procedures and animal treatment were in accordance with the Principles of Laboratory Animal Care issued by the National Society for Medical Research (authorization no. 006235 from French ministry of agriculture). For cutaneous wounds, the selection of the closure device depends essentially on the depth of the wound. For superficial lacerations, use of suture, adhesive tapes, and cyanoacrylate adhesives such as 2-octyl-cyanoacrylate, *N*-butul-2-cyanoacrylate-methacryloxysulfolane, *N*-butyl-2-cyanoacrylate) are the current methods of choice in humans.1c For deep wounds, closure suturing is the clinical gold standard (Figure 1).[1a,b, 12] Indeed, cyanoacrylate adhesives provoke local tissue reaction (toxicity and/or inflammation) and form layers that prevent tissue direct contact (Figure 1).

**Figure 1:**
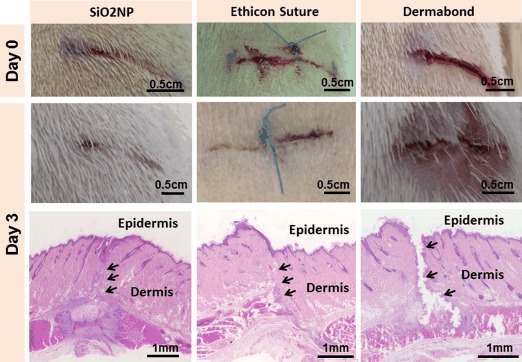
In vivo comparison of repair by SiO2NP nanobridging, by suturing, and by cyanoacrylate glue of full-thickness dorsal skin injury in a Wistar rat model. A drop of SiO2NP solution was put onto a wound edge with a brush and the two wound edges were gently pressed into contact for about a minute. The other wounds were closed with a non-resorbable suture (Ethicon 4/0) and 2-octyl cyanoacrylate (Dermabond). At day 3 post-surgery, no wound leakages, infection, or inflammatory reactions were observed after nanobridging with SiO2NP. The rat skin closure achieved with nanoparticles and the suture were comparable. For the cyanoacrylate glue, the wound edges were not bonded correctly. Histological sections were stained with Hematoxylin-Phloxin-Saffron stain.

In contrast, nanoparticles should not lead to formation of a rigid macroscopic barrier, and thanks to their size should not affect substantially the natural wound healing process. We therefore aimed for repair by nanobridging of full thickness cutaneous incisions and compared resulting healing with that of sutured incisions in Wistar rats. Because the healing depends of the thickness of the skin and of the local skin state of tension,[1a, 13] we investigated the efficiency of nanobridging in two different sites: the thin abdominal skin and the thick dorsal skin and results were comparable.

In Figure 1, a dorsal wound of 1.5 cm in length and 3 mm in depth was nanobridged by Stöber silica (SiO2NP and results compared to a standard suture by non-resorbable clinical thread (4/0, Ethicon) and commercial cyanoacrylate glue (Dermabond). Nanoparticle solutions were spread with a brush (*n*=6) or a micropipette (*n*=5) on one edge of the wound and two edges were brought together manually and pressed into contact (Supporting Information, Figure S1, Movie S1). By using a micropipette, we could vary the volume of nanoparticle solution spread (from 2 μL to 15 μL). Excess solution, which rose to the wound surface, was removed with a compress. The wound edges were maintained in contact manually for less than one minute after which time the wound has closed. For all of the animals, wounds did not reopen during the follow up. The macroscopic results evidenced no pathological inflammation or necrosis (Figure 1). Figure 2 shows results for deep dorsal wound closure achieved with metabolizable[10] iron oxide nanoparticles.

**Figure 2:**
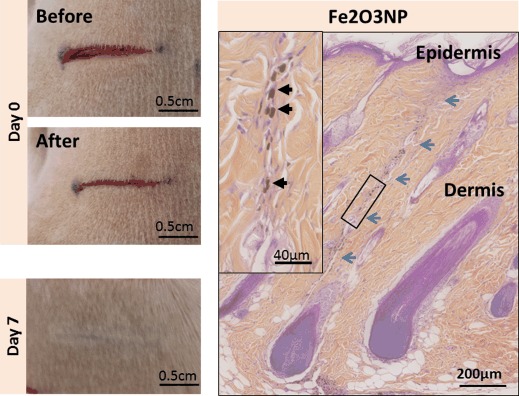
Skin wound closure with Fe2O3NP solution. After skin injury, 4 μL of Fe2O3NP solution was soaked onto one edge of the wound. A thin and aesthetic scar was observed. At day 7 post-injury, the histological sections stained with Hematoxylin-Phloxin-Saffron stain evidenced the site of the injury as a very thin line (blue arrows). Magnification (inset) of this area revealed a normal repair process and some particle aggregates along the wound closure.

For all of the tested nanoparticles, scars were aesthetic (Figure 1 and 2), a feature which bodes well for many areas of skin surgery. Furthermore, nanobridging allows easily repositioning and adjusting wound edges to obtain an optimal alignment. Repositioning is in principle possible for suturing, but it requires removal of suture by trained personnel and increases operation time, and adds to local trauma that delays healing.

The presence of nanoparticles applied by brush or micropipette does not modify the first stages of healing process, namely vascular clot formation and inflammation that prevent bleeding and remove cells and dying tissue.[14] As for sutures, the granulation tissue formed a new connective matrix serving as a migration structure for the cells (Figure 1). For Fe2O3 particles, Hematoxylin-Phloxine-Saffron staining reveals the presence of small amount of aggregates (Figure 2). Controlling particle aggregation is important. Indeed, when powders of silica nanoparticle rather than solutions were spread, the particle agglomerates limit wound closure and healing (Supporting Information, Figure S2).

Cauterization, sutures, or hemostatic sealants can treat surface lacerations of soft and wet tissues deeply penetrated by blood such as liver, spleen, or kidney.[2, 11a–d,g] However, use of these techniques for deep wounds closure is very challenging. A 1.5 cm long and 6 mm deep horizontal incision on a right hepatic rat lobe was performed with a scalpel. In control experiments, the mechanical pressure did not yield any permanent hemostasis in the absence of nanoparticle solution and lead to hemorrhage and death. To repair, SiO_2_ or Fe_2_O_3_ nanoparticle solutions were deposited to the bleeding injury area with a pipette. The two edges of the wound were brought manually together and kept in contact. After about 1 min hemostasis was complete, and the injury stayed closed (Supporting Information, Movie S2). The rats were monitored during the acute post-surgery, and no bleeding syndrome was detected (*n*=3). At day 3 post-surgery, stereo-macroscopic observation of the liver showed a thin scar tissue (Figure 3). Histological studies revealed the presence of thin granulation tissue between the two edges of the injury. Nanobridging not only assured hemostasis, biliostasis, and wound closure, but liver function was also not affected by the application of nanoparticle solutions. Alat and Asat enzymes were in normal range, respectively, 26 U L^−1^ and 81 U L^−1^ before surgery and 24 U L^−1^ and 74 U L^−1^ 3 days after repair by SiO2NP. The total bilirubin was in the normal range (1.4 μmol L^−1^ and 1.5 μmol L^−1^, respectively, before and 3 days post-surgery).

**Figure 3:**
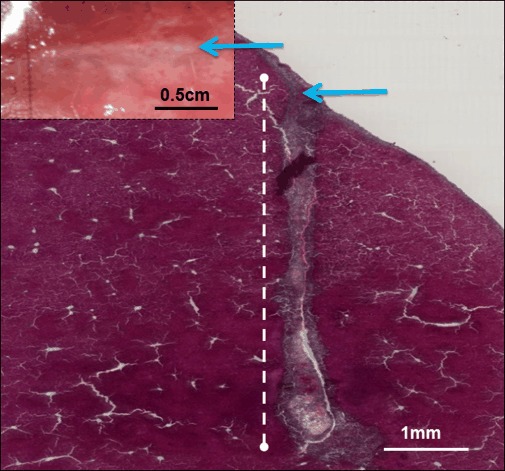
Liver injury repair with Ludox TM50 silica nanoparticles. A 6 mm deep horizontal incision was performed with a scalpel on a right hepatic rat lobe and nanoparticle solution was deposited to the bleeding injury with a pipette, then the edges of the wound were brought together. After about 1 min, hemostasis was complete. Three days post-injury, macroscopic examination of the liver surface showed a thin fibrotic line at the site of the injury (Inset, blue arrows). Histological cross-section of the wound (Hematoxylin-Phloxin-Saffron stain), showing the formation of tissue repair from the liver surface (blue arrow) and along the wound (white dotted line).

For hemostasis after hepatectomy, we propose employing nanoparticles to firmly attach membranes onto bleeding liver section. To illustrate the potential of such an approach, we used a poly(vinyl alcohol) (PVA) membrane with a surface coated with silica nanoparticles.[15] PVA film was swollen in phosphate buffer saline solution. The coating was realized by spreading SiO2NP powder on a surface of the swollen film. The unattached silica particles were removed by gently shaking the film. A ventral midline laparotomy (5 cm) was performed on a Wistar rat. The right hepatic lobe was exposed and resection of 2/3 of the lobe was totally transversally cut and the coated membrane was lightly pressed for few seconds against the bleeding section (Figure 4). Hemostasis was immediately obtained. After 15 min of monitoring, the abdominal wall was closed with a Vicryl 4/0 and the rat was monitored during the acute post-surgery without any evidence of a bleeding syndrome. As shown in Figure 4, neither pathologic inflammation nor bleeding was observed at the site of injury three days after surgery. In control experiments, no hemostatic seal could be achieved with PVA membrane in the absence of SiO2 coating.

**Figure 4:**
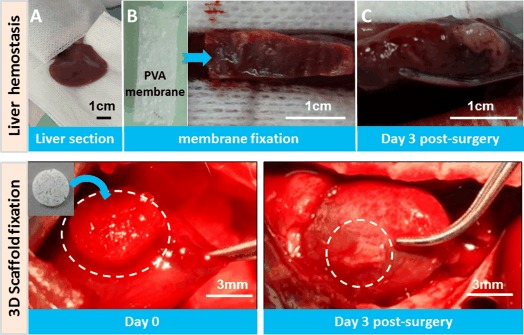
Hemostasis and medical device securing to tissues. Top: Hemostasis after liver resection. A) The right lobe of the rat liver was sectioned (A) and a wet-PVA membrane coated by SIO2NP nanoparticles was spread to cover the liver section (B). The bleeding stopped within 30 seconds. After 3 days, the liver was explanted and the membrane was still present (C). Bottom: Securing a construct to a beating heart. A solution of Ludox TM50 silica nanoparticles was applied with a brush onto the surface of a beating rat heart. A porous and biodegradable 3D-polysaccharide scaffold was then simply put onto the coated area and it firmly bonded to the beating heart surface (left; Supporting Information, Movie S3). The scaffold was still attached to the heart surface after 3 days.

For a membrane fixed onto liver tissue to stop bleeding, organ motions are limited. In many clinical situations, it is important to secure membranes, medical devices, or tissue engineering constructs to organs that undergo important contractions, such as the beating heart.[8a, 16] The application of adhesives is thus much more demanding and, when possible, suturing or cyanoacrylate glues are employed, despite their toxicity and the difficulty of applying in wet conditions. To check whether the adhesion brought by nanoparticles can withstand stringent in vivo conditions and prevent device slipping, we evaluated the ability of nanobridging to fix a scaffold onto the beating heart of rats. Rats were thus anesthesied and a tracheal intubation and mechanical ventilation were performed. The thorax was opened, and a drop of the silica Ludox TM50 was spread on the surface of the heart with a brush. A 3D-scaffold of 6 mm in diameter made of a porous polysaccharide biodegradable hydrogel optimized for cell therapy[16] was brought into contact with the surface coated by nanoparticles and stayed firmly fixed resisting heart contractions and the wet environment (Figure 4; Supporting Information, Movie S3). After 3 days, the thorax was re-opened and the 3D-scaffold was still visible on the heart (Figure 4). Macroscopic evaluation did not show any sign of inflammation and as expected the degradation of the polysaccharide scaffold had begun.

In summary, we have demonstrated that rapid and strong adhesion by aqueous solutions of nanoparticles can be advantageously used in very different clinical situations. For skin wounds, remarkably aesthetic healing was obtained and repair procedure does not require any specific preparation or training. Bleeding control and tissue repair by nanobridging shown herein in the case of liver could be used on spleen, kidney, heart, and lung surgeries. When tight sealing is needed, nanobridging could complement anastomosis and classical suturing procedures. The possibility of securing medical devices could open new applications in repair and regenerative medicine. From the standpoint of chemistry, the principle illustrated herein is not limited to silica and iron oxide nanoparticles, and they are many possible choices of sizes, forms, and surface chemistry. In particular, nanoparticles with intrinsic biological effects, such as silver nanoparticles for skin infection or drug delivery systems, could provide useful options. Translation to clinical practice will require careful safety and toxicity investigations. A better understanding of biological mechanisms of the adhesion by nanobridging will guide the design of future-generation tissue adhesives.
