# Supplementary material for: Organ Repair, Hemostasis, and In Vivo Bonding of Medical Devices by Aqueous Solutions of Nanoparticles
Source: Angew Chem Int Ed Engl. 2014 Apr 16;53(25):6369–73. doi: 10.1002/anie.201401043 (PMC4320763; doi:10.1002/anie.201401043)
Supplement: Supplementary file 3 [file anie0053-6369-sd3.pdf]

Angewandte Chemie
Eine Zeitschrift der Gesellschaft Deutscher Chemiker

Supporting Information
© Wiley-VCH 2015
69451 Weinheim, Germany

Organ Repair, Hemostasis, and In Vivo Bonding of Medical Devices by Aqueous Solutions of Nanoparticles**

Anne Meddahi-Pellé, Aurélie Legrand, Alba Marcellan, Liliane Louedec, Didier Letourneur, and Ludwik Leibler**

anie_201401043_sm_miscellaneous_information.pdf
anie_201401043_sm_beating_heart.mp4
anie_201401043_sm_liver_injury_repair.mp4
anie_201401043_sm_skin_injury_repair.mp4

Experimental section

Nanoparticles preparation

Silica SiO2NP nanoparticles were prepared using Stöber et al. method.[1] In particular 600 mL of absolute ethanol and 36 mL of ammonium hydroxide solution (35 wt.% in water) were added to a round bottom flask and stirred for 5 min. 18 mL of TEOS were then quickly poured and the resulting solution was stirred overnight at room temperature. Silica particles were retrieved by centrifugation (7600 rpm, 45 min) and washed with absolute ethanol and followed by four cycles of centrifugation- dispersion. Silica particles were eventually air dried over 6 hours at 80°C. Particles characterization was performed using dynamic light scattering (DLS) and transmission electron microscopy (TEM). The particles hydrodynamic radius (DLS) was 80 nm and the polydispersity index 15%. The radius determined from TEM images analysis was about 50 nm (Supporting Information, Fig. S4). The particles were dispersed in milli-Q waters at 30 wt%. Silica Ludox® TM-50 water solutions with concentration of 52 wt% of silica particles at pH 9 with particle radius of about 15 nm was purchased from Aldrich and used as received.

Iron oxide Fe2O3NP nanoparticle solutions were prepared using commercially available (NanoArc® purchased from Alfa Aesar) magnetic Fe_2O_3 nanoparticles of 20-40 nm diameter and surface area equal to 30-60 m^2/g. In particular, 0.5067 g of Fe_2O_3 nanoparticles were dispersed in 8 mL of milli-Q water via ultrasonication for 5 minutes. The dispersion was subsequently transferred in a glass reactor equipped with a glass anchor-shaped stirrer that was preloaded with 100 mL of 0.02 M citric acid and was left under mechanical stirring overnight. Following the collection of the particles in a flask, they were decanted using a magnet and washed three times with milli-Q water. The citrated particles were re-dispersed in 12 mL milli-Q water via ultrasonication and were peptized with 40 µL of 35 % w/w NH_4OH aqueous solution to obtain a 42.2 g/L concentration of the initial Fe2O3 particles. The pH of the solution measured using a pH meter paper was found to be between 7 and 7.5. Supporting Information Fig. S5 shows TEM image of Fe2O3NP nanoparticles.

[1] W. Stöber W., A. Fink, E. Bohn, *J. Coll. Interface Sci.* **1968**, *26*, 62–69.

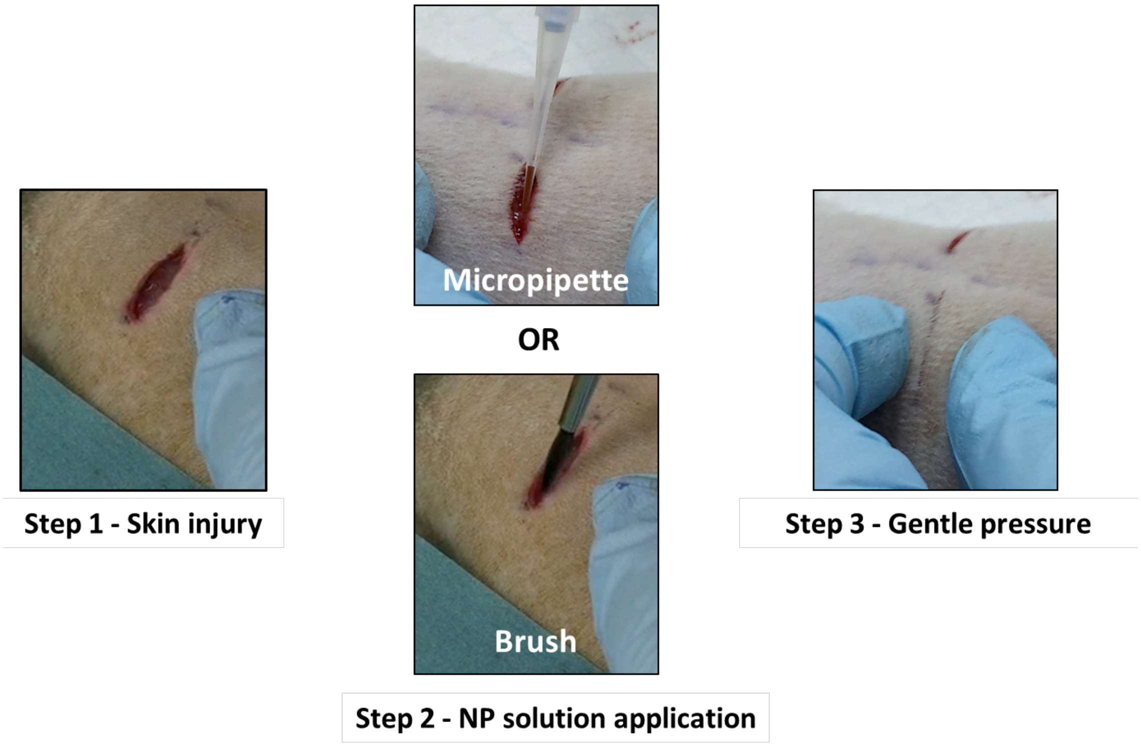

Figure S1: Application of nanoparticle solution for skin wound closure. Step 1: After skin injury, a nanoparticle solution was spread at the edge of the wound with a brush or with a micropipette to better control the spread volume. Gentle mechanical pressure was applied (step 3). The skin wound was closed within a minute (step 4).

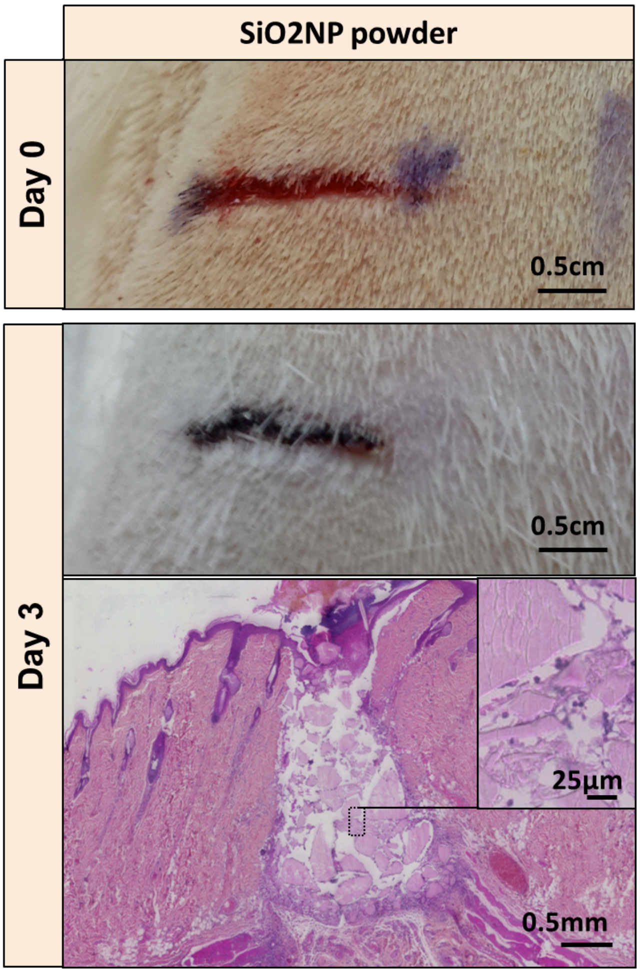

Figure S2: Silica nanoparticule powder was applied to the wound. At day 3 the scar was still visible and the cross-section stained with Hematoxylin Phloxin Saffron showed the skin defect, which was filled by silica agregates. At high magnification (Inset), few cells were observed around the aggregates that cover a large area, and the granulation tissue had not developed.

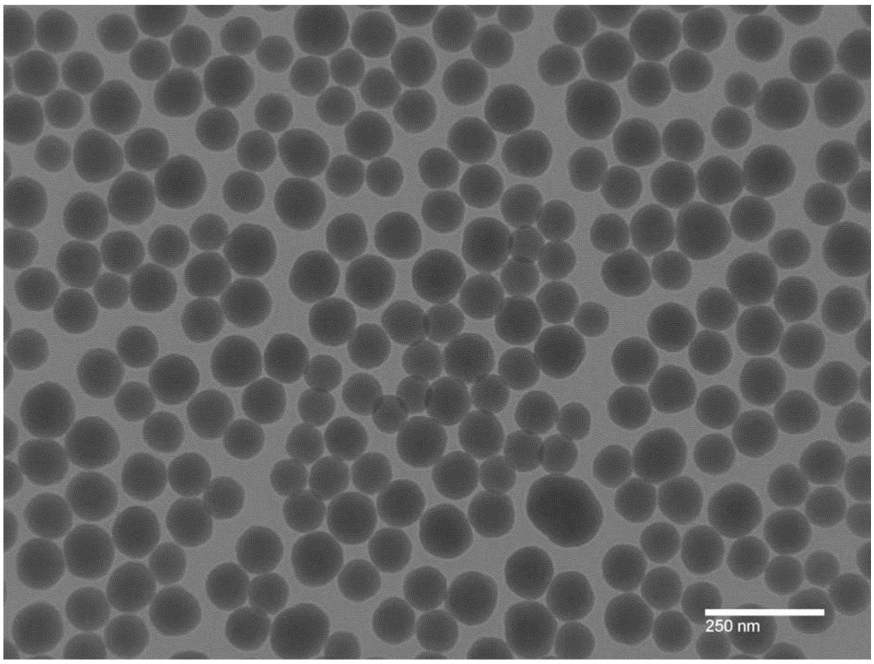

Figure S3: TEM micrograph of SiO2NP nanoparticles.

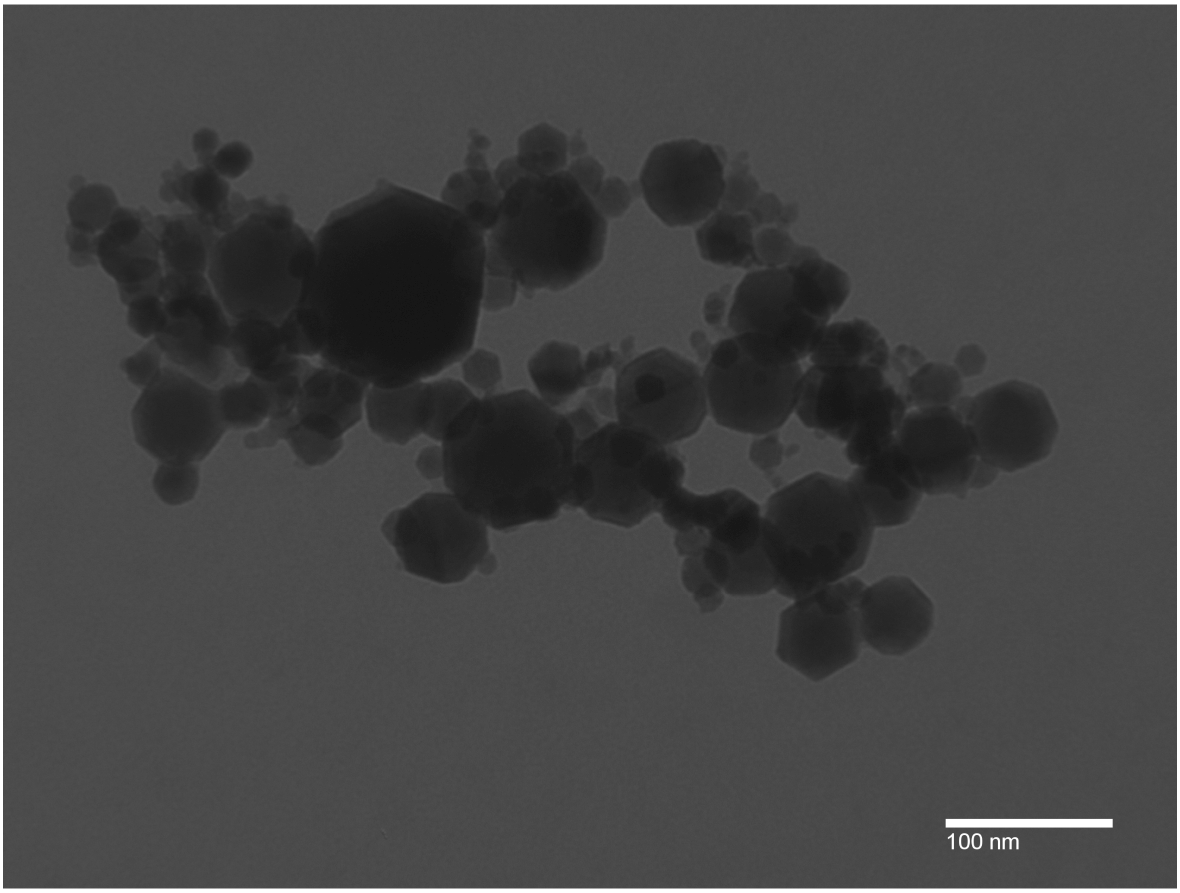

Figure S4: TEM micrograph of Fe2O3NP nanoparticles.
